# Cystic fibrosis transmembrane conductance regulator (CFTR) and autophagy: hereditary defects in cystic fibrosis *versus* gluten-mediated inhibition in celiac disease

**DOI:** 10.18632/oncotarget.27037

**Published:** 2019-07-09

**Authors:** Luigi Maiuri, Valeria Raia, Mauro Piacentini, Antonella Tosco, Valeria Rachela Villella, Guido Kroemer

**Affiliations:** ^1^ Department of Health Sciences, University of Eastern Piedmont, Novara, Italy; ^2^ European Institute for Research in Cystic Fibrosis, San Raffaele Scientific Institute, Milan, Italy; ^3^ Department of Translational Medical Sciences, Pediatric Unit, Regional Cystic Fibrosis Center, Federico II University Naples, Naples, Italy; ^4^ Department of Biology, University of Rome "Tor Vergata", Rome, Italy; ^5^ National Institute for Infectious Diseases, IRCCS ‘L. Spallanzani’, Rome, Italy; ^6^ Equipe 11 labellisée Ligue Nationale contre le Cancer, Centre de Recherche des Cordeliers, Paris, France; ^7^ INSERM U1138, Centre de Recherche des Cordeliers, Paris, France; ^8^ Université Paris Descartes/Paris V, Sorbonne Paris Cité, Paris, France; ^9^ Metabolomics and Cell Biology Platforms, Institut Gustave Roussy, Villejuif, France; ^10^ Pôle de Biologie, Hôpital Européen Georges Pompidou, AP-HP, Paris, France; ^11^ Department of Women's and Children's Health, Karolinska University Hospital, Karolinska Institute, Stockholm, Sweden; ^12^ Suzhou Institute for Systems Medicine, Chinese Academy of Sciences, Suzhou, China

**Keywords:** CFTR, cystic fibrosis, autophagy, celiac disease, transglutaminase 2

## Abstract

Cystic Fibrosis (CF) is the most frequent lethal monogenetic disease affecting humans. CF is characterized by mutations in cystic fibrosis transmembrane conductance regulator (CFTR), a chloride channel whose malfunction triggers the activation of transglutaminase-2 (TGM2), as well as the inactivation of the Beclin-1 (BECN1) complex resulting in disabled autophagy. CFTR inhibition, TGM2 activation and BECN1 sequestration engage in an ‘infernal trio’ that locks the cell in a pro-inflammatory state through anti-homeostatic feedforward loops. Thus, stimulation of CFTR function, TGM2 inhibition and autophagy stimulation can be used to treat CF patients. Several studies indicate that patients with CF have a higher incidence of celiac disease (CD) and that mice bearing genetically determined CFTR defects are particularly sensitive to the enteropathogenic effects of the orally supplied gliadin (a gluten-derived protein). A gluten/gliadin-derived peptide (P31–43) inhibits CFTR in mouse intestinal epithelial cells, causing a local stress response that contributes to the immunopathology of CD. In particular, P31–43-induced CFTR inhibition elicits an epithelial stress response perturbing proteostasis. This event triggers TGM2 activation, BECN1 sequestration and results in molecular crosslinking of CFTR and P31-43 by TGM2. Importantly, stimulation of CFTR function with a pharmacological potentiator (Ivacaftor), which is approved for the treatment of CF, could attenuate the autophagy-inhibition and pro-inflammatory effects of gliadin in preclinical models of CD. Thus, CD shares with CF a common molecular mechanism involving CFTR inhibition that might respond to drugs that intercept the "infernal trio". Here, we highlight how drugs available for CF treatment could be repurposed for the therapy of CD.

## INTRODUCTION

The proteostasis network ensures intracellular homeostasis in spite of endogenous and exogenous perturbations that lead to changes in protein conformation and abundance [1]. Autophagy is a major player in the proteostasis network, allowing for the regulated turnover of large protein aggregates and even entire organelles. Moreover, components of the autophagy machinery dynamically interact with multiple signaling pathways to optimize cellular adaptation to cell-autonomous or environmental stress signals [2, 3].

Cystic fibrosis (CF) transmembrane conductance regulator (CFTR) is a unique member of the ATP-binding cassette (ABC) transporters family that acts as a cyclic adenosine monophosphate (cAMP)-regulated anion channel mediating chloride/bicarbonate transport across epithelia [4, 5]. Emerging evidence indicates that CFTR does not merely function as an anion channel but that it also orchestrates the proteostasis network. Indeed, CFTR operates in a context-specific dynamic system of interactor proteins that is connected to, and influenced by, the proteostasis network [6–8]. Functional perturbation of CFTR can result from inherited loss-of-function mutations that cause CF [5] or pharmacological inhibition of CFTR channel activity [8]. In either case, CFTR inhibition leads to a major derangement of cellular proteostasis. CFTR malfunction increases the generation of reactive oxygen species (ROS) that induce post-translational modifications of transglutaminase 2 (TGM2), a versatile multifunctional enzyme that catalyzes several post-translational modifications of target proteins [9, 10]. TGM2 undergoes small ubiquitin like-modifier (SUMOylation), a post-translational modification that inhibits TGM2 ubiquitination leading to persistent high TGM2 protein levels and TGM2 activation as the result of permissive elevated Ca2+ levels [11]. Activated TGM2 targets a plethora of substrates, among which the essential autophagy protein Beclin 1 (BECN1) [6–8], that is essential for autophagosome formation [2]. BECN1 targeting by TGM2 causes its dislodgement, as well as that of several BECN1 interactors, away from the endoplasmic reticulum, leading to its functional sequestration in intracellular aggregates [6–8]. Inactivation of the protein complex organized around BECN1 has two major negative effects on cellular proteostasis. First, the functional sequestration of phosphatidylinositol 3-kinase catalytic subunit type 3 (PIK3C3) and of UV radiation resistance-associated-gene (UVRAG), two major components of the BECN1 complex, negatively impacts on intracellular trafficking in CF epithelial cells, as it reduces the availability of phosphatidyl-inositol-3-phosphate (PtdIns3P) at early endosomes and perturbs endosomal fusion/maturation and trafficking [6, 12, 13]. Second, BECN1 inactivation disables autophagy, leading to defective autophagosome formation and accumulation of the autophagic substrate sequestosome 1 (SQSTM1) at endosomal level with subsequent reduced availability of the small GTPase RAB5 and RAB7, which are essential for endosomal maturation [14]. In addition, the ubiquitin-binding-protein SQSTM1 accumulates at the plasma membrane (PM) and favors the disposal of several surface proteins, including epidermal growth factor receptor (EGFR) and CFTR itself [7, 8]. Importantly, defective CFTR results in the activation of the innate immune system at the mucosal surface, as it leads to TGM2-mediated sequestration of the anti-inflammatory peroxisome proliferator-activated receptor-γ (PPARγ), and of nuclear factor kappa-light-chain-enhancer of activated B cells (NF-κB) inhibitor alpha (NFKBIA) within histone-deacetylase 6 (HDAC6)+/vimentin+ intracellular aggresomes. The neutralization of NFKBIA results in nuclear translocation of the pro-inflammatory transcription factor of NF-κB, thus increasing the transactivation of genes coding for pro-inflammatory cytokines [7, 11]. Thus, downstream of the CFTR defect and ROS generation, TGM2 activation can function as a rheostat of the post- translational network.

### CFTR, TGM2 and autophagy

Notably, CFTR, TGM2 and autophagy are engaged in a feed-forward loop, meaning that CFTR dysfunction activates TGM2 and disables autophagy, while the inhibition of TGM2 and the restoration of autophagy re-establishes CFTR function at the cell surface. This intimate connection between CFTR, TGM2 and autophagy, can be conceived as a common platform for the surveillance of cellular homeostasis. In this perspective, CFTR can be viewed as a major sensor of stress, that alerts the autophagy machinery when a stressful event risks to perturb cellular physiology. Accordingly, CFTR malfunction in macrophages of CF patients compromises the autophagy-mediated clearance of Pseudomonas aeruginosa and Burkholderia cepacia [15, 16]. Defective bacterial clearance can be reverted by restoring CFTR function at the cell surface. In addition, the interaction of functional CFTR with caveolin-1 (CAV1) in airways is required to avoid excessive Toll-like receptor 4 (TLR4) signaling upon exposure to bacterial products [17].

The correction of deficient proteostasis by means of so-called proteostasis regulators constitutes an emerging strategy for palliating CFTR malfunction arising from loss-of-function mutations in the CFTR gene [18–20]. The approximately 2000 CFTR mutations have been categorized in 6 classes according to their impact on the synthesis (class I), processing (class II), gating (class III), conductance (class IV), quantity (amount) (class V) or recycling (class VI) of the CFTR protein [5]. Among these mutants, the most frequent one is the class II F508del-CFTR mutant that occurs in 70 to 90% of CF patients either in a heterozygous or homozygous form. Mutation-specific drugs have been approved by regulatory instances (such as the Food and Drug Administration, FDA, and the European Medicine Agency, EMA) and directly target the mutated CFTR protein to increase its PM expression (correctors) or improve its ion transport function (potentiators) [5]. In contrast, proteostasis regulators aim at targeting the cellular environment in which mutant CFTR traffics and functions [18–20]. Proteostasis regulators interrupt the feed-forward loop between CFTR, TGM2 and autophagy to reestablish autophagy flux that is deranged by, but can also impact on, the CFTR defect. In line with this evidence, the proteostasis regulator cysteamine, a TGM2 inhibitor that prevents TGM2-mediated BECN1 sequestration, can reestablish autophagic flux and restore the function of the F508del-CFTR mutant at the epithelial surface, both in patients and in mice models bearing a similar CFTR mutation [18–20]. Interestingly, the beneficial effects of cysteamine on both CFTR function and autophagy persist for several weeks after cysteamine withdrawal. Thus, restoration of proteostasis results in transient homeostasis before the system again loses its balance. Of note, in CF patients bearing class II CFTR mutations, treatment with epigallocatechin-gallate (EGCG, an inhibitor of the autophagy-inhibitory acetyl transferase EP300) [21] can prolong the beneficial effects of cysteamine with respect to autophagy induction and restore CFTR function in nasal respiratory epithelial cells [18, 19]. Preclinical studies involving F508del-CFTR mice indicate that the aforementioned combination treatment (cysteamine plus EGCG) loses its capacity to restore CFTR function in a Becn1 haploinsufficient (Becn1+/-) background [19]. Thus, autophagy is required for sustaining a functional CFTR at the cell surface.

## CF AND CD: NEW MECHANISM OF CONNECTION

### CF features

CF is best known for its respiratory phenotype, which results from increased viscosity of the mucus in the lung, increased pulmonary infections, and chronic inflammation [5, 22]. Thus, defective CFTR function ultimately drives inflammation, persistent and untreatable bacterial colonization and recurrent chest infections, mostly by *Pseudomonas aeruginosa*, *Staphylococcus aureus* and *Burkholderia cepacia*, causing chronic progressive lung disease with bronchiectasis and alveolar destruction culminating in respiratory insufficiency [23]. Beyond its respiratory manifestations, CF is a systemic disease because CFTR is expressed in, and is relevant to the function of, many tissues, including the small and large intestines, pancreas, the biliary tree, the male reproductive tract and sweat glands [5, 24]. Gastrointestinal symptoms of CF are not only attributable to thick and sticky mucus in the intestine and in pancreatic ducts that lead to exocrine pancreatic insufficiency, but are also due to a constitutive intestinal inflammation owing to CFTR malfunction [5, 24]. Preclinical evidence indicates that defective autophagy downstream to CFTR malfunction is pivotal for driving the disease phenotype at the intestinal level. Thus, CF mice feed with a standard diet usually succumb to intestinal obstruction after weaning unless they are orally treated with cysteamine to restore autophagy and to consequently rescue CFTR function [18].

### CD pathogenesis

The intestine of CF patients is exposed to a particularly high antigenic load due to the exocrine pancreatic insufficiency. Indeed, patients suffering from CF may exhibit increased levels of antibodies against dietary antigens, increased fecal calprotectin levels, alteration of the intestinal microbiota, as well as increased intestinal permeability [24]. In particular, a ~4% prevalence of positive anti-TGM2-IgA autoantibodies, a serological marker of celiac disease (CD), has been reported in several cohorts of CF patients [25–28]. Moreover, the prevalence of CD is three times higher in CF patients than in the general population [28]. Moreover, mice that are CFTR deficient due to the CFTR knock-out mice or the F508del-CFTR knock-in mutation differed from wild type mice in thus far that they developed signs of ileal inflammation when they were fed with the gluten component gliadin. Thus, inherited CFTR malfunction favors gliadin responsiveness [28]. Based on these results, the question arises as to whether CFTR might be conceived as a protective mucosal shield that usually prevents CD. CD manifests as a permanent intolerance to dietary proteins from wheat, rye and barley, occurring in ~1 % of individuals worldwide. In the CD intestine, the ingestion of gluten proteins including gliadin results into an adaptive immune response against gliadin-derived peptides with an autoimmune component [29, 30]. It is known that some peptide fractions from gliadin are particularly pathogenic. Thus, the peptide P31-43 must ignite an innate immune response in epithelial cells, while the peptide P57-68 can induce cognate immune responses by T cells and antibodies in a subset of genetically susceptible individuals bearing the human leukocyte antigen (HLA) DQ2/DQ8 [31–33]. In intestinal epithelia from celiac patients, P31-43 enters the endosomal compartment, triggers TGM2 activation, perturbs endosomal maturation and trafficking, and activates the NF-κB pathway [34], which are all features reminiscent of those occurring in respiratory epithelia from CF patients.

### CFTR and P31-43 peptide relationship

Recently, we have demonstrated that CFTR and autophagy are major players in the pathogenesis of CD. In intestinal epithelial, P31-43 encounters CFTR in clathrin+ vesicles and binds to, and reduces the ATPase activity of the nuclear-binding-domain-1 (NBD1) of CFTR, thus impairing CFTR function [28]. Similarly, to the inherited CFTR defect associated with CF, the gliadin-induced CFTR malfunction occurring in CD results in the activation of TGM2 and perturbs the autophagy machinery. Indeed, P31-43-mediated inhibition of CFTR drives TGM2 activation that covalently crosslinks P31-43, CFTR and TGM2 in a trimolecular complex, thus amplifying the detrimental effects of gliadin. This results in reduced activity of the BECN1 complex with reduced availability of PtdIns3P and UVRAG at the endosomal level together with SQSTM1 accumulation. In addition, CFTR malfunction suffices to increase the production of interleukin-15 (IL15), a pro-inflammatory cytokine critical for CD pathogenesis [29, 32, 33, 35–37], as the result of TGM2-mediated NF-κB activation, exactly as this occurs in CF epithelia [28].

In sum, important alterations of cellular activity such as TGM2 activation and autophagy inhibition related to CFTR malfunction (due to mutations in CF or gliadin-derived peptide in CD), represent an "infernal trio" [38] (characterized by three alterations: inhibition of CFTR, activation of TGM2, disablement of autophagy) that eliminate a loops of cellular stress.

### Drug repurposing in CD?

Of note the CFTR chloride channel function constitutes a potential therapeutic target in CD. Thus, maintaining CFTR in an open conformational state by means of pharmacological potentiators (such as the FDA/EMA-approved drug Ivacaftor, formerly called VX-770), can avoid P31-43 binding to NBD1, thus preventing its inhibitory effect on CFTR. As a consequence, CFTR potentiators can protect intestinal epithelial cells from P31-43 induced stress response as they control TGM2 activation, restore the function of the BECN1 complex, prevent the accumulation of SQSTM1 and restore endosomal trafficking [28]. Importantly, CFTR potentiators prevent IL15 upregulation and control P31-43 driven immune activation [28]. These effects were initially observed in cultured human epithelial cells and then reproduced *in vivo*, in gliadin-sensitive BALB/C mice, as well as in non-obese diabetic (NOD) mice which are particularly susceptible to oral challenge with gliadin [39–42]. In these models of gliadin sensitivity, pretreatment with the CFTR potentiator Ivacaftor, a drug approved for the treatment of CF patients bearing particular *CFTR* mutations [5, 43], prevents the gliadin-induced suppression of the CFTR-mediated chloride current (as observable in the small intestine mounted in Ussing chambers responding to the CFTR stimulator forskolin) and controls the gliadin-induced intestinal inflammation. Importantly, Ivacaftor also controls the adaptive immune response, and instead promotes a tolerogenic response to gliadin in peripheral blood mononuclear cells (PBMC) from celiac patients that are co-cultured with gliadin-challenged intestinal epithelial cells [28].

Based on these findings, other additional strategies might intercept central mechanisms of both CD and CF, targeting the aforementioned ‘infernal trio’ [38]. First, it may be possible to use other CFTR potentiators including Vrx-532 [28] or genistein [44] that would be expected to act similarly to Ivacaftor [28]. Second, the inhibition of TGM2 with cysteamine or other, more specific agents that are currently in development [6, 11, 28, 45–49], should restore BECN1 and autophagy, thereby protecting CftrF508del mice from the increased responsiveness to gliadin [50]. Finally, autophagy stimulation could be achieved by inhibition of the acetyltransferase EP300 (examples: aspirin, epigallocatechin gallate, EGCG, and spermidine) [21, 51, 52], neutralizing BECN1 inhibitory proteins from the BCL2 family (examples: ABT737, navitoclax, venetoclax) [53] or inhibitors of the mechanistic target of rapamycin complex-1 (mTORC1; examples: rapamycin, tacrolimus) [54].

It is important to note that each of the aforementioned strategies for the treatment of CD (for which one of the primary causes apparently is the gluten-mediated CFTR inhibition) has been successfully applied to CF (which is due to inherited loss-of-function CFTR mutations): i) Ivacaftor has initially been designed for improving the function of specific CFTR mutants in CF and is right now FDA and EMA-approved for the treatment of CF [43, 55–58]; ii) cysteamine can be used to inhibit TGM2 and is able to restore CFTR protein and function at the PM of cultured cells from patients with the CFTRF508del/F508del mutation [18, 19, 47]; iii) the autophagy activator EGCG, combined with cysteamine, improves and prolongs its rescuing effect [19, 58]. Altogether, these findings illustrate the clinical feasibility of tackling the ‘infernal trio’.

## CONCLUSIONS

**Figure 1:**
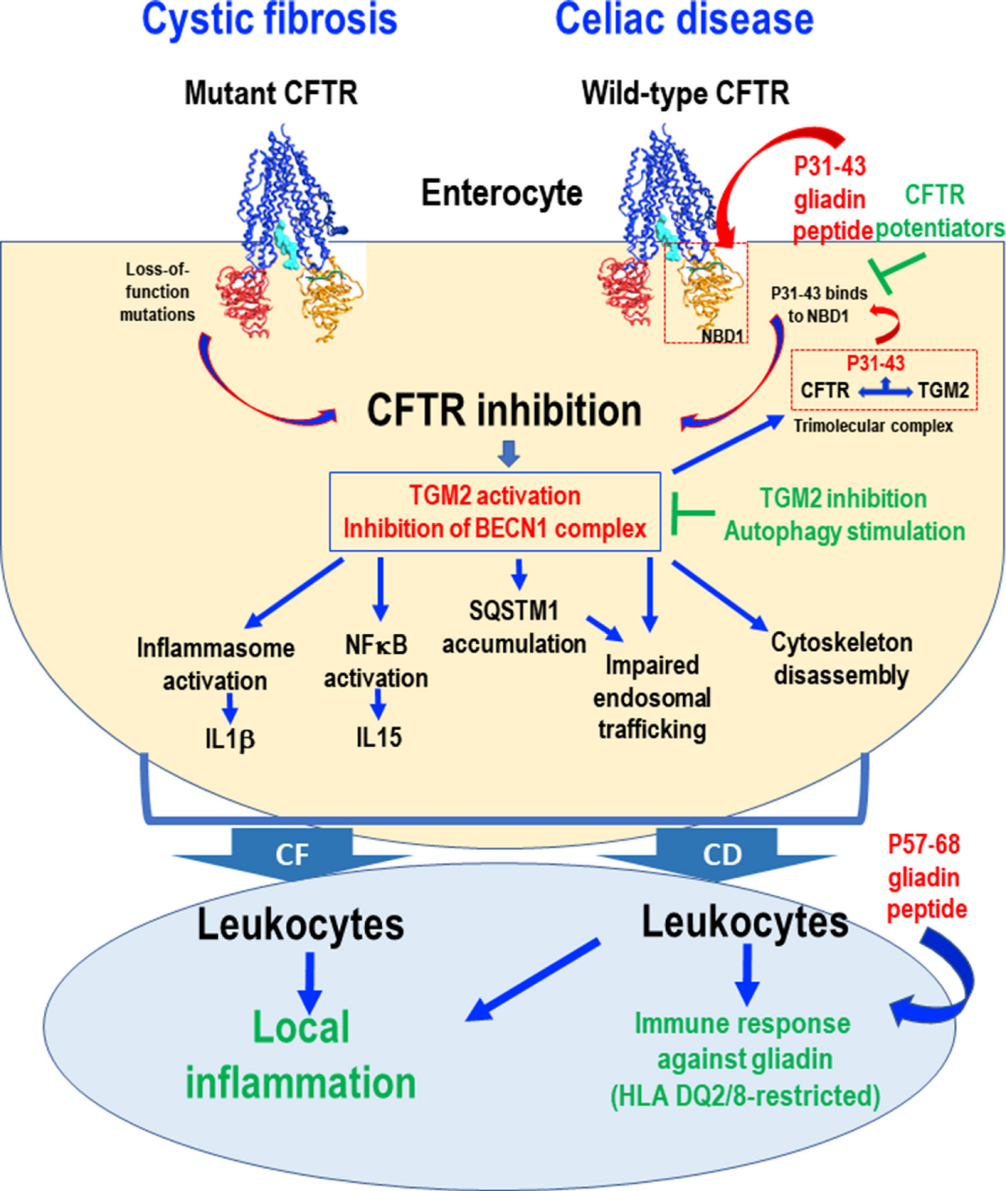
Schematic view of common pathogenic events downstream of CFTR inhibition in cystic fibrosis (CF) and celiac disease (CD). Loss-of-function mutations in the CFTR gene cause CFTR inhibition in CF. In the intestine from CD patients, the gliadin-derived P31-43 peptide interacts with, and binds to, specific residues of the NBD1 domain of CFTR, if the domain is in its inactive conformation, thus competing with ATP binding and blocking CFTR function. In both CF and CD epithelial cells, CFTR inhibition disrupts cellular proteostasis through two effects (i) transglutaminase-2 (TGM2) activation and (ii) BECN1 complex inhibition. In CD, TGM2 accessorily is recruited to a tripartite complex that stabilizes P31-43 binding to CFTR, thus worsening CFTR inhibition. In both conditions, CFTR inhibition leads to impaired endosomal trafficking, cytoskeleton disassembly, inflammasome activation resulting in interleukin-1β (IL1β) secretion, NF-κB activation and consequent interleukin-15 (IL15) production. Stressed enterocytes stimulate local inflammation in both CF and CD. In the gut from CD patients, this ignites the immune responses against gliadin, in particular P57-68, in a context of HLA-DQ2/DQ8. This pathogenic cascade can be interrupted by CFTR potentiators that prevent P31-43 binding to CFTR or by reconstitution of cellular proteostasis by TGM2 inhibition or BECN1 complex activation.

In conclusion, CFTR can be inhibited in two apparently different diseases, in CF, where CFTR is mutated, and in CD, where CFTR is inhibited by gluten/gliadin-derived peptides [59] (Figure 1). In both conditions, CFTR inhibition ultimately compromises autophagy, thus reducing the capacity of cells to withstand stress and maintain tissue homeostasis. Downstream of CFTR, TGM2 plays a major role to connect CFTR malfunction and autophagy inhibition in a vicious cycle, as TG2 inhibition can interrupt this feed-forward loop [9, 10, 49]. As a perspective, stimulation of CFTR function by pharmacological potentiators, inhibition of TGM2, as well as reactivation of autophagy by suitable drugs, may be used for the treatment of both CF and CD.

It might be important to explore the possibility to combine such close-to-etiological treatments with suitable life style interventions to avoid excessive gluten/gliadin uptake, as well as with non-specific measures designed to dampen inflammation and to improve the gut barrier function.

As a final note, it should be mentioned that new findings suggest that CFTR dysfunction contributes to the pathogenesis of other inflammatory state affecting epithelia [60, 61]. Thus, it is tempting to speculate, yet remains to be demonstrated, that impaired autophagy and CFTR malfunction are often associated among each other to drive the phenotype of a variety of human diseases.

## Abbreviations

CFTR: cystic fibrosis transmembrane conductance regulator
CF: cystic fibrosis
CD: celiac disease
HLA: human leukocyte antigen
ROS: reactive oxygen species
TGM2: tissue transglutaminase 2
BECN1: Beclin 1
NBD1: nuclear binding domain 1
FDA: Food and Drug Administration
VX-770: Ivacaftor
NF-κB: nuclear factor kappa B
IL-15: interleukin-15
NOD mice: non-obese diabetic mice
